# The Presence of Comorbid ADHD and Anxiety Symptoms in Autism Spectrum Disorder: Clinical Presentation and Predictors

**DOI:** 10.3389/fpsyt.2018.00717

**Published:** 2018-12-20

**Authors:** Einat Avni, Esther Ben-Itzchak, Ditza A. Zachor

**Affiliations:** ^1^The Autism Center, Department of Pediatrics, Assaf Harofeh Medical Center, Tel Aviv, Israel; ^2^Bruckner Center for Research in Autism Spectrum Disorder, Communication Disorder Department, Ariel University, Ariel, Israel; ^3^Department of Pediatrics, Sackler Faculty of Medicine, Tel Aviv University, Tel Aviv, Israel

**Keywords:** autism (ASD), ADHD (attention deficit and hyperactivity disorder), anxiety, autism severity, adaptive skills, cognitive abilities

## Abstract

High rates of attention deficit/hyperactivity disorder (ADHD) and anxiety symptoms have been documented in autism spectrum disorder (ASD), and have been associated with social and adaptive impairments. The study examined the frequency of clinically elevated ADHD and anxiety symptoms in an ASD group in comparison to a non-clinical group, compared the clinical presentation in the ASD group with and without ADHD and anxiety, assessed which child and familial variables add to the severity of Inattention, Hyperactivity/Impulsivity (HI), and anxiety symptoms, and evaluated whether having clinically elevated ADHD and/or anxiety symptoms adds to the prediction of adaptive functioning in ASD. The study included 260 participants diagnosed with ASD (mean age: 7.5 ± 1.1), using standardized tests. The rate of clinically elevated ADHD and anxiety symptoms in ASD was 62.7 and 44.6%, respectively, and symptom severity was significantly greater than the non-clinical sample. The entire population was divided into four subgroups: ASD alone, ASD+ADHD, ASD+anxiety, ASD+ADHD+anxiety, based on the parental behavioral questionnaire. The ASD alone group showed less severe autism symptoms in comparison to the other groups. Having ASD+ADHD symptoms was associated with greater impairments in socialization adaptive skills. Only the group with ASD+ADHD+anxiety was associated with poorer daily living adaptive skills. Regression analyses for prediction of ADHD and anxiety symptoms revealed that being a female and having lower adaptive skills scores predicted higher Inattention severity; being older, having better cognition, and more severe Restrictive Repetitive Behavior symptoms predicted more severe HI symptoms; being older and having more severe social impairments predicted higher anxiety scores. A regression analysis for the prediction of adaptive skills revealed that in addition to cognition and autism severity, the severity of Inattention symptoms added to the prediction of overall adaptive skills. In light of these findings, clinicians should diagnose these comorbidities in ASD early on, and provide effective interventions to reduce their negative impact on functioning, thereby improving outcome.

## Introduction

Autism spectrum disorder (ASD) is a neurobehavioral disorder defined by social- communication deficits and restricted and repetitive behaviors that are typically detectable in early childhood and continue into adulthood (1). ASD-specific behaviors have been found to negatively impact various aspects of the lives of individuals with this disorder (2). The highly heterogeneous nature of ASD is often reflected in the child's characteristics, including clinical variability in the severity of autism symptoms, cognitive ability, and language skills (3). In addition, substantial individual differences are apparent with regard to the occurrence of co-morbidities such as attention-deficit/hyperactivity disorder (ADHD) and anxiety (4–9).

ADHD is a common neurodevelopmental disorder with a prevalence estimated at 7.2% in the general population (10). ADHD is characterized by a persistent pattern of inattention and/or hyperactivity and impulsivity that is pervasive across settings and leads to various degrees of functional impairment (11, 12). Children and adolescents with ASD have shown high rates of ADHD symptoms (16–85%) (5–7, 9) and overlap between ASD and ADHD symptoms has been described as well (13). However, the recognition that the diagnoses of ADHD and ASD can occur together has been formalized only in the DSM-5 (1). Previous studies have examined the co-morbidity of ADHD in ASD and described more severe autism symptoms (14), higher rates of cognitive impairment (10), more deficits in adaptive skills (2, 15) and lower quality of life (10) in individuals with ASD and ADHD in comparison to ASD alone. These studies were all cross-sectional and groups were not based on having an ADHD diagnosis but rather on ADHD symptom severity. These studies used different ADHD rating scales and behavioral checklists, completed by different informants (parents, teachers or both) (2, 10, 14, 15). Using standardized tests, Sprenger et al. reported a greater severity of autistic symptoms in children with ASD and ADHD symptoms, especially in the social interaction domain, than in those with ASD alone (14). Sikora et al. (2) found greater impairment in adaptive functioning among children with ASD and clinically significant ADHD symptoms in comparison to children with ASD and fewer ADHD symptoms. In contrast, Ashwood et al. (15) found significant associations between reduced adaptive functioning and autism symptoms, but not with ADHD symptoms.

The prevalence of anxiety disorders in the general population is 6.5% (16), ranging from 3 to 24% in various studies (17). Anxiety is among the most common mental health problems in ASD (4, 8). Similarly to ADHD, higher frequencies of anxiety difficulties (11–84%) have been reported in children with ASD in comparison to the general population (18) and up to 40% are diagnosed with at least one DSM anxiety disorder at some point in their lives (19, 20). Anxiety disorders may include features of excessive fear and related behavioral disturbances (1). Some studies have noted that ASD is a vulnerability for stress and anxiety (20). In contrast, according to some cross-sectional research, anxiety may be an underlying cause of several symptoms of ASD. For example, anxiety affects the stereotypical and rigid behaviors (21) and social functioning difficulties (22) that children with an ASD often face. Furthermore, anxiety in children with ASD has a negative impact on adaptive functioning, daily living skills (DLS), and relationships with peers, teachers, and family (23, 24). For example, Factor et al. (25) found more social difficulties in youth with ASD and anxiety symptoms in comparison to youth with only ASD. Most of the studies that examined ASD and anxiety were cross-sectional ones, and anxiety symptoms were assessed using different anxiety scales.

Rates of anxiety in the presence of ADHD in the general population range from 13 to 50%, and these comorbidities together are associated with greater risk of long term impairments than children with either condition (26). However, only a few studies have examined the frequency or the clinical impact of anxiety in children with both ASD and ADHD. Craig et al. (27) found that individuals with ASD and ADHD had higher frequency of anxiety, as compared to individuals with only ASD. Since having ASD and ADHD is already known to negatively affect functioning (2, 10, 14, 15), it is assumed that having anxiety in addition to ADHD will have additive negative impact on children with ASD. Other studies have suggested that some of the anxiety symptoms overlap with ASD symptoms (21, 22).

In light of this, it is possible that the clinical presentation in ASD would not be worsened by having anxiety symptoms. Therefore, is it important to further investigate the contribution of having anxiety symptoms in ASD with comorbid ADHD on various aspects of the clinical presentation.

The study has several aims. First, to evaluate the frequency of ADHD and anxiety symptoms in the participants diagnosed with ASD and to compare them to non-clinical standardized samples. Second, to compare the clinical presentation in the ASD participants with and without ADHD and anxiety, in terms of autism symptom severity, adaptive skills, and cognitive ability. Third, to assess the relationships between the severity of the ADHD and anxiety symptoms and the autism severity and adaptive functioning. The fourth aim is to assess which variables (including age, sex, parental educational attainment, cognition, autism severity, and adaptive skills) add to the severity of Inattention, Hyperactivity/Impulsivity, and anxiety symptoms individually. The fifth aim is to assess whether having ADHD and/or anxiety symptoms adds to the prediction of adaptive skills functioning in the ASD participants, beyond the role of the child's other characteristics.

We have several hypotheses:

1. Clinically elevated ADHD and anxiety symptoms frequently occur in children with ASD. Rates of ADHD and anxiety in the ASD group will be significantly higher compared to the non-clinical standardized samples.
2. The participants with ASD and clinically elevated ADHD and/or anxiety symptoms, and particularly those with ASD+ADHD+anxiety, will present with more severe autism symptomatology, lower cognition, and lower adaptive skills, in comparison to those without these comorbid symptoms.
3. Higher cognition, more severe autism symptoms and lower adaptive skills will be associated with and predict more severe ADHD and anxiety symptoms.
4. More severe ADHD and anxiety symptoms in ASD will be associated with and predict lower adaptive functioning

This study is the first to examine a large group diagnosed with ASD and two major comorbidities and assesses their relationship to the clinical presentation.

## Methods

### Measures

#### Autism Diagnostic Interview-Revised (ADI–R)

A semi-structured interview administered to parents, designed to make a diagnosis of autism according to DSM-IV criteria. Diagnosis of ASD was made on meeting the cutoff points of the ADI-R algorithm scores in the three domains: social interaction, communication, and restricted repetitive behavior (RRB) (28). Autism severity was assessed based on the level of the scores; higher scores in each ADI subdomain reflected more severe autism symptoms.

#### Autism Diagnosis Observation Schedule (ADOS)

A semi-structured, interactive schedule conducted by skilled professionals designed to assess social and communicative functioning in individuals who may have ASD (29). The scores of each of the ADOS subdomains, social affect (SA) and RRB, were used for the calculation of each subdomain severity score using the SA and RRB-calibrated severity scale (CSS) (30). Higher scores in the ADOS subdomains reflect more severe autism symptoms.

#### Wechsler Preschool and Primary Scale of Intelligence—Third Edition (WPPSI-IIIHEB)

An intelligence test for children aged 2:6–7:3, conducted by skilled psychologists. It consists of 4 subscales: Verbal IQ, Performance IQ, Processing Speed and Full Scale IQ. All the indices including Full Scale IQ were represented as standard scores: mean = 100; *SD* = 15 (31).

#### Wechsler Intelligence Scale for Children IV (WISC-IV)

A measure of intellectual ability and cognitive processing for children aged 6:0–16:11, conducted by skilled psychologists. It has ten core and five supplemental subtests. The subtests can be clustered into composite quotients for four indices: Verbal Comprehension, Perceptual Reasoning, Working Memory and Processing Speed. All the indices including full scale IQ were represented as standard scores: mean = 100; *SD* = 15 (32).

#### Vineland Adaptive Behavior Scales (VABS)

A standardized caregiver interview designed to assess adaptive behaviors in children between the ages of birth and 18 years. The VABS yields a composite score as well as scores in four domains: Communication, Daily Living Skills, Socialization, and Motor Skills (until the age of 6 years), each of which yields a standard score (33). Higher scores in the subdomains reflect better functioning.

#### Conners' Rating Scales–Revised: Long Form (CRS-R:L)

Parent and teacher questionnaires normed for children and adolescents 3–17 years old that assess ADHD symptomatology (i.e., inattention, hyperactivity, impulsivity), anxiety, and other co-morbid behaviors. Estimates of symptom severity are obtained using T-scores (mean = 50, *SD* = 10); higher T-scores reflect greater psychopathology (34). For ADHD symptoms, we used three scales of the parents' questionnaire: ADHD Index, DSM-IV inattentive and the DSM-IV hyperactive. For anxiety symptoms, we used the anxious-shy scale (8 items; i.e., easily frightened, afraid of people, afraid of new situations, afraid to be alone, overly attached to caregivers, and so forth). Only scores ≥60 in the corresponding scale were considered significant.

### Procedure and Participants

The study was conducted at a national tertiary center for autism that is involved in diagnosis, treatment, and research in ASD. Data from clinical records were collected on 1,143 children who were assessed for a possible ASD diagnosis between January 2010 and December 2015. The children underwent a comprehensive evaluation including a neurological assessment by a pediatric developmental neurologist, and behavioral and cognitive evaluations conducted by a skilled interdisciplinary team. Information about previous diagnoses was obtained from previous assessments performed in other centers. Assessment of ASD was obtained using standardized tests, the Autism Diagnosis Interview-Revised (ADI-R) (28) and the Autism Diagnosis Observation Schedule (ADOS) (29), and meeting criteria for ASD based on DSM-IV (35) or DSM-5 criteria (1), depending on the year of diagnosis. The ADOS and ADI-R were administered by child neurologists and Masters' level psychologists, all of whom established at least 80% concurrence with research reliable psychologists on three consecutive administrations. Inclusion criteria were having an ASD diagnosis and being in the age range of 5–12. Out of the 1,143 children who were evaluated at the center, only 264 matched our study's criteria for inclusion. Participants with diagnoses of specific genetic syndromes and sensory impairments (*n* = 4) were excluded from the study. The final cohort included 260 participants (228 males, 32 females), with a mean age of 7 years and 5 months (*SD* = 1 year and 1 month). All the participants were Caucasians, and Hebrew was the spoken language.

The results for the ADI-R were available for all 260 participants and for the ADOS for 255 participants. Cognitive and developmental abilities (IQ/DQ) were assessed using the Wechsler Preschool and Primary Scale of Intelligence (WPPSI) (31) or the Wechsler Intelligence Scale for Children IV (WISC-IV) (32), according to the child's age and language level, and results were available for 232 participants. The assessment of adaptive skills was obtained using the Vineland Adaptive Behavior Scales (VABS) (33) and was available for all 260 participants. All the children underwent a thorough neurological assessment, which included a semi-structured interview for comorbidities, such as ADHD, anxiety, oppositional defiant disorder, and mood disturbances. The severity of ADHD symptomatology (i.e., inattention, hyperactivity, and impulsivity) and anxiety were assessed using the Conners' Rating Scales–Revised: Long form (CRS-R:L) (34). T-score ≥60 supported significant symptoms in the correlating scale.

In the current research, although parents' and teachers' CRS scores are presented and compared, only the parents' questionnaire was used to define the groups, in order to avoid the impact of ADHD medications given during the school day, which may affect the severity of ADHD symptoms as reported by the teachers. The study participants were divided into four groups based on the ADHD index and anxiety scales of the Conners Parents' Rating Scales–Revised: Long (CPRS-R:L) questionnaire (results were available for all 260 participants): the first group, with an ADHD index <60, and anxiety <60, was designated as ASD alone (*N* = 68); the second group, with an ADHD index ≥60, and anxiety <60, was designated as ASD with ADHD (ASD+ADHD; *N* = 76); the third group, with an ADHD index <60 and Anxiety ≥60, was designated as ASD with anxiety (ASD+anxiety; *N* = 29); the fourth group, with an ADHD index ≥60 and anxiety ≥60, was designated as ASD with anxiety and ADHD (ASD+ADHD+anxiety; *N* = 87).

### Data Analysis

To compare the CPRS subdomain mean scores of the ASD study group with data on a non-clinical group taken from the CRS manual, a one sample *T*-test analysis was used.

To examine the effect of the coder who completed the CRS (parent/teacher), a one-way MANOVA with a repeated measure of coder was performed for the Inattention, HI, and Anxiety subdomain scores.

Comparisons of demographic data between the four examined groups (ASD alone, ASD+ADHD, ASD+anxiety, ASD+ADHD+anxiety) included sex using non-parametric chi-square analysis, age using one-way ANOVAs, and parental ages and educational attainment using one-way MANOVAs. Comparisons between the four examined groups of the dependent variables—including IQ/DQ scores, ADI-R subdomain (social interaction, communication and RRB) scores, ADOS subdomain (SA- and RRB-CSS) scores, and VABS composite and subdomain (Communication, DLS, Socialization) scores—were performed using one-way ANOVAs and MANOVAs. When a MANOVA yielded a significant group effect, individual ANOVAs were performed for each of the examined variables. In the analyses that yielded a significant group effect, *post-hoc* Scheffe tests were performed.

Next we examined the correlations between CPRS subdomain (IA, HI and anxiety) scores with IQ scores and VABS, ADI-R, and ADOS subdomain scores using Pearson correlation analyses.

Finally, to identify the variables that contributed to the explained variance in the CPRS subdomains, three five-step hierarchical linear regression analyses were performed with CPRS Inattention, HI, and Anxiety scores as the dependent variables.

To identify the variables that contributed to the explained variance in the VABS composite scores, another five-step hierarchical linear regression analysis was performed.

### Compliance With Ethical Standards

All procedures performed in this study were approved by the ethical standards of the institutional research committee and with the 1964 Helsinki declaration and its later amendments or comparable ethical standards. The Helsinki committee of the medical center waived the need for informed consent, as the study was retrospective and all the data were derived from the clinical charts.

## Results

### Prevalence of ADHD and Anxiety Symptoms

The frequency of clinically elevated ADHD symptoms as reported by the parents (CPRS ADHD index ≥60) in the entire ASD group was 62.7%. “Inattention” symptoms (CPRS DSM-IV inattentive ≥60) were reported in 67.3% of the participants, and “hyperactivity/impulsivity” symptoms (CPRS DSM-IV hyperactive ≥60) in 56.5% of the participants.

The frequency of clinically elevated anxiety symptoms as reported by the parents (CPRS anxious-shy ≥60) in the entire ASD group was 44.6%.

At the time of evaluation at the Autism Center, 74.5% (*n* = 194) of the study participants were not previously diagnosed with ASD. Among those participants, 52.9% had at least one previous medical, neurodevelopmental and/or behavioral diagnosis. The most prevalent diagnosis was ADHD (*n* = 61; 31.4%) followed by developmental delay (*n* = 48, 29.3%) and language disorder (*n* = 19, 9.8%). Among the group with a previous ASD diagnosis (25.5%, *n* = 66), 10 participants (15%) received an ADHD diagnosis in addition to the principal diagnosis of ASD. Comparing the CPRS subdomain mean scores of the ASD group to the mean scores of the non-clinical group (ages 3–12) taken from the CRS manual revealed significantly higher scores in the Inattention [*t*_(258)_ = 26.33, *p* < 0.01], HI [*t*_(258)_ = 23.53, *p* < 0.01] and Anxiety [*t*_(258)_ = 15.40, *p* < 0.01] subdomains for the current study's ASD group.

### Parents' and Teachers' Reports of ADHD and Anxiety Symptoms

As presented in Table 1, parent and teacher CRS ratings were compared, and yielded a significant coder effect [*F*_(3, 247)_ = 38.53, *p* < 0.001, η^2^ = 0.32]. For the clinically elevated ADHD symptoms (Inattention and Hyperactivity/Impulsivity), the teachers' CRS scores were lower than the parental CRS. In contrast, for clinically elevated anxiety symptoms, teachers' CRS scores were higher than the parents'. For the clinically elevated CRS Hyperactivity/Impulsivity and the CRS anxiety scores, the effect size was medium. However, for the clinically elevated CRS Inattention scores the effect size was small.

**Table 1:** Mean standard scores of parent and teacher ratings on CRS subdomains.

	**Parents**	**Teacher**	***F*_**(1, 249)**_**	**η^2^**
CRS IA	65.08 (11.28)	61.26 (11.06)	24.73^***^	0.09
CRS HI	62.59 (11.94)	58.18 (11.26)	31.69^***^	0.11
CRS Anxiety	61.40 (13.75)	68.25 (13.37)	44.14^***^	0.15

CRS, Conners' rating scales; IA, inattention; HI, hyperactivity/impulsivity.

*** *p < 0.001*.

### Clinical Presentation of ASD With and Without Clinically Elevated ADHD and Anxiety Symptoms

We compared the four defined groups based on the CPRS ADHD index scores and the anxious-shy scores. The male:female ratio did not differ significantly among the groups in a non-parametric chi-square analysis [χ${}_{\left(3\right)}^{2}$ = 3.18, *p* > 0.05]. As presented in Table 2, parental ages at pregnancy and educational attainment in the four examined groups were not significantly different using one-way MANOVAs. Only the ANOVA for the child's age yielded a significant main effect, however *post-hoc* Scheffe analysis did not reveal any significant difference between each pair of the groups.

**Table 2:** Mean scores and standard deviation of demographic variables in the ASD alone, ASD+ADHD, ASD+anxiety, and ASD+ADHD+anxiety groups.

	**ASD**	**ASD+ADHD**	**ASD+Anxiety**	**ASD+ADHD+Anxiety**	***F***	**μ^2^**
	***N* = 68**	***N* = 76**	***N* = 29**	***N* = 87**		
Age	6:10 (1:11)	7:4 (1:11)	7:10 (2:1)	7:7 (1:11)	2.83^*^	0.03
Maternal age	30:4 (4:6)	29:10 (4.00)	30:0 (4:6)	29:8 (4:11)	0.26	0.00
Paternal age	33:8 (6:1)	32:4 (5:0)	32:6 (5:2)	32:4 (5:5)	1.04	0.01
Maternal education	15:6 (2:7)	15:8 (2:4)	14:8 (2:4)	15:4 (3:1)	0.88	0.00
Paternal education	14:8 (2:7)	15:1 (2:6)	14:10 (4:0)	15:1 (3:6)	2.2	0.00

ASD, autism spectrum disorder; ADHD, attention-deficit/hyperactivity disorder.

* *p < 0.05*.

#### Cognitive Ability

Regarding cognitive ability, using a one-way ANOVA, no group main effect was found (Table 3).

**Table 3:** Mean scores and standard deviation of IQ and VABS domains in the ASD alone, ASD+ADHD, ASD+anxiety, and ASD+ADHD+anxiety groups.

	**ASD**	**ASD+ADHD**	**ASD+Anxiety**	**ASD+ADHD+Anxiety**	***F***	**μ^2^**
	***N* = 68**	***N* = 76**	***N* = 29**	***N* = 86**		
Cognition	90.22 (17.14)	90.41 (22.72)	83.93 (17.77)	84.58 (19.92)	1.71	0.02
VABS communication	86.90 (13.11)	83.10 (16.0)	86.10 (14.12)	81.33	2.20^∧^	0.02
VABS DLS	83.23 (9.59)	79.56 (13.02)	78.24 (8.89)	76.03 (12.05)	5.10^**^	0.06
VABS socialization	80.73 (11.67)	73.90 (11.85)	75.00 (14.08)	70.83 (10.78)	9.25^***^	0.10
VABS composite scores	81.59 (10.07)	76.99 (12.03)	78.34 (10.01)	74.57 (10.63)	5.32^***^	0.06

VABS, adaptive behavior scales; ASD, autism spectrum disorder, ADHD, attention-deficit/hyperactivity disorder; DLS, daily living skills.

*** p < 0.001,

** p < 0.01,

∧ *p < 0.1*.

#### Adaptive Skills

Regarding adaptive behavior skills, using a one-way MANOVA, a group effect was found [*F*_(9, 765)_ = 3.68, *p* < 0.001, η^2^ = 0.04]. When examining each subdomain separately, the daily living skills and the socialization subdomains yielded a significant group main effect with medium effect size (Table 3). For the DLS subdomain, the ASD alone group had significantly higher scores than the ASD+ADHD+anxiety group (*p* < 0.001); for the socialization subdomain, the ASD alone group had significantly higher scores than the ASD+ADHD group (*p* < 0.01) and the ASD+ADHD+anxiety group (*p* < 0.05) (*post-hoc* Scheffe).

#### Autism Severity

We then compared autism severity in the four examined groups, using the ADI-R scores, which were based on parental reports, and the ADOS scores, which were based on professional assessments. Comparing ADI-R subdomain scores between the four examined groups using a one-way MANOVA revealed a significant group effect [*F*_(9, 768)_ = 4.99, *p* < 0.001, η^2^ = 0.05]. Separately examining each ADI-R subdomain using one way ANOVAs yielded a significant group effect in all ADI-R subdomains (Table 4). For the social interaction subdomain, the ANOVA yielded a large effect size. The ASD alone group had lower scores than the other three groups (0.001 < *p* < 0.05) and the ASD+ADHD group had significantly lower scores than the ASD+ADHD+anxiety group (*p* < 0.05). For the communication and RRB subdomains, the ANOVAs yielded medium effect sizes. For the communication subdomain, only the ASD alone group had significantly lower scores than the ASD+ADHD+anxiety group (*p* < 0.001). For the RRB subdomain, the ASD alone group had significantly lower scores than the other three examined groups (0.001 > *p* < 0.01) (*post-hoc* Scheffe tests).

**Table 4:** Mean scores and standard deviation of ADOS and ADI-R subdomains in the ASD alone, ASD+ADHD, ASD+anxiety, and ASD+ADHD+anxiety groups.

	**ASD**	**ASD+ADHD**	**ASD+ Anxiety**	**ASD+ ADHD+Anxiety**	**F**	**μ^2^**
					**ASD±ADHD**	
ADOS–SA-CSS	8.14 (1.68)	7.40 (2.13)	8.00 (2.27)	8.11 (1.76)	2.38^∧^	0.03
ADOS-RRB-CSS	8.33 (2.29)	8.40 (1.92)	7.55 (2.57)	8.26 (1.82)	1.26	0.01
ADI-R SI	9.59 (5.26)	12.33 (5.70)	13.34 (6.00)	15.10 (5.72)	12.43^***^	0.13
ADI-R communication	8.96 (4.00)	10.85 (4.33)	10.44 (4.66)	12.19 (4.81)	6.8^***^	0.07
ADI-R RRB	4.23 (2.56)	5.78 (2.67)	6.21 (2.68)	6.52 (2.84)	9.70^***^	0.10

ADOS, autism diagnosis observation schedule; ADI-R, autism diagnostic interview-revised; ASD, autism spectrum disorder; ADHD, attention-deficit/hyperactivity disorder; SA, social affect; RRB, restricted repetitive behavior; CSS, calibrated severity scale; SI, social interaction.

*** p < 0.001,

∧ *p < 0.1*.

Comparing the ADOS-CSS between the defined groups, the one-way MANOVA yielded a significant group main effect [*F*_(6, 502)_ = 2.16, *p* < 0.05, η^2^ = 0.02], however, the separated ANOVAs for each subdomain did not yield a significant main effect.

### Correlations Between ADHD and Anxiety Symptom Severity and the Clinical Presentation

We examined the correlations of all three CPRS subdomains–Inattention (IA) Hyperactivity/Impulsivity (HI) and Anxiety, with VABS, ADI-R, and ADOS CSS subdomain scores (Table 5). We used continuous scores for these analyses. CPRS subdomains correlated negatively and significantly with all VABS subdomains and composite scores (except for CPRS HI and VABS communication subdomain) and positively with all ADI-R subdomains. Most correlations were in the medium range. The correlations of CPRS IA and ADI-R RRB, CPRS HI and VABS DLS and CPRS anxiety and VABS communication were all in the small range.

**Table 5:** Correlations of CPRS subdomains scores with VABS, ADI–R, and ADOS subdomain scores.

	**VABS**	**VABS DLS**	**VABS**	**VABS**	**ADI-R SI**	**ADI-R**	**ADI-R RRB**	**ADOSSA**	**ADOS RRB**
	**communication**		**socialization**	**composite**		**communication**		**CSS**	**CSS**
CPRS IA	−0.24^***^	−0.28^***^	−0.27^***^	−0.30^***^	0.23^**^	0.26^***^	0.15^**^	−0.05	0.05
CPRS HI	−0.10	−0.17^**^	−0.30^***^	−0.20^***^	0.27^***^	0.27^***^	0.38^***^	−0.01	0.03
CPRS anxiety	−0.13^*^	−0.23^***^	−0.30^***^	−0.22^*^	0.34^***^	0.28^***^	0.28^***^	−0.08	−0.09

CPRS, Conners' parents' rating scales; VABS, adaptive behavior scales; ADI-R, autism diagnostic interview–revised; ADOS, autism diagnosis observation schedule; IA, inattention, HI, hyperactivity/impulsivity; DLS, daily living skills; SI, social interaction; RRB, restricted repetitive behavior; SA, social affect; CSS, calibrated severity scale.

*** p < 0.001,

** p < 0.01,

* *p < 0.5*.

### Factors Predicting the Severity of ADHD and Anxiety Symptoms

To identify variables that contributed to the variance in the CPRS scores, we applied three five-step hierarchical linear regression analyses using the CPRS Inattention, Hyperactivity/Impulsivity, and Anxiety scores as the dependent variables. We used continuous scores for these analyses. The independent variables of age and sex were entered in the first step, maternal education in the second step, cognitive scores in the third step, the ADI-SI, ADI Communication, and ADI RRB scores in the fourth step, and the VABS composite score in the fifth step.

#### Inattention

As presented in Table 6, the first regression analysis with the CPRS Inattention scores as the dependent variable explained 12.3% of the variance. In the final step, when all predictors were entered together, only sex and VABS composite scores were significant independent predictors over and above all the other variables entered.

**Table 6:** Linear regression model for the CPRS inattentive scores.

**Variable**	**B**	**SE.B**	**β**	***R*^**2**^**	**Δ*R*^**2**^**
**Step 1**				0.04^**^	0.04^**^
Sex	6.47	2.24	0.19^**^		
Age	−0.01	0.03	−0.02		
**Step 2**				0.04^*^	0.00
Sex	6.47	2.24	0.19^**^		
Age	−0.01	0.03	−0.02		
Maternal education	0.12	0.28	0.03		
**Step 3**				0.04^*^	0.00
Sex	6.23	2.26	0.18^**^		
Age	−0.01	0.03	−0.02		
Maternal education	0.16	0.27	0.04		
Cognition	−0.03	0.04	−0.06		
**Step 4**				0.09^***^	0.05^**^
Sex	6.76	2.23	0.20^***^		
Age	−0.02	0.03	−0.05		
Maternal education	0.10	0.28	0.02		
Cognition	−0.01	0.04	−0.02		
ADI-R–SI	0.18	0.18	0.09		
ADI-R–communication	0.40	0.22	0.16^*^		
ADI-R–RRB	0.04	0.31	0.01		
**Step 5**				0.12^***^	0.03^**^
Sex	6.62	2.20	0.20^***^		
Age	−0.03	0.03	−0.07		
Maternal education	0.14	0.28	0.03		
Cognition	0.04	0.04	0.06		
ADI-R–SI	0.06	0.18	0.03		
ADI-R–communication	0.30	0.22	0.12		
ADI-R–RRB	0.04	0.30	0.01		
VABS composite scores	−0.23	0.09	−0.22^**^		

CPRS, conners' parents' rating scales; ADI-R, autism diagnostic interview–revised; SI, social interaction; RRB, restricted repetitive behavior; VABS, adaptive behavior scales.

*** p < 0.001,

** p < 0.01,

* *p < 0.5*.

#### Hyperactivity/Impulsivity

The second regression analysis with CPRS IH scores as the dependent variable (Table 7) explained 18% of the variance. In the final step, when all predictors were entered together, only age and ADI-R-RBB scores were significant independent predictors over and above all the other variables entered.

**Table 7:** Linear regression model for the CPRS HI scores.

**Variable**	**B**	**SE.B**	**β**	***R*^**2**^**	**Δ*R*^**2**^**
**Step 1**				0.03^*^	0.03^*^
Sex	1.05	2.36	0.03		
Age	0.08	0.03	0.17^**^		
**Step 2**				0.03^*^	0.00
Sex	1.07	2.36	0.03		
Age	0.08	0.03	0.17^**^		
Maternal education	0.16	0.30	0.03		
**Step 3**				0.04^*^	0.01
Sex	1.37	2.38	0.04		
Age	0.08	0.03	0.17^**^		
Maternal education	0.11	0.30	0.02		
Cognition	0.04	0.04	0.07		
**Step 4**				0.18^***^	0.14^***^
Sex	3.04	2.23	0.09		
Age	0.07	0.03	0.15^**^		
Maternal education	−0.05	0.28	−0.01		
Cognition	0.08	0.04	0.14^**^		
ADI-R–SI	0.08	0.18	0.04		
ADI-R–communication	0.25	0.22	0.09		
ADI-R–RRB	1.33	0.31	0.32^***^		
**Step 5**				0.18	0.00
Sex	3.00	2.23	0.08		
Age	0.07	0.03	0.15^**^		
Maternal education	−0.04	0.28	−0.01		
Cognition	0.10	0.04	0.17^**^		
ADI-R–SI	0.04	0.18	0.02		
ADI-R–communication	0.21	0.22	0.08		
ADI-R–RRB	1.32	0.31	0.32^***^		
VABS composite score	−0.08	0.09	−0.07		

CPRS, conners' parents' rating scales; HI, hyperactivity/impulsivity; ADI-R, autism diagnostic interview–revised; SI, social interaction; RRB, restricted repetitive behavior; VABS, adaptive behavior scales.

*** p < 0.001,

** p < 0.01,

* *p < 0.5*.

#### Anxiety

The third regression analysis with CPRS anxiety scores as the dependent variable (Table 8) explained 18.0% of the variance. In the final step, when all predictors were entered together, only age and ADI-R social interaction scores were significant independent predictors over and above all the other variables entered.

**Table 8:** Linear regression model for the CPRS anxiety scores.

**Variable**	**B**	**SE.B**	**β**	***R*^**2**^**	**Δ*R*^**2**^**
**Step 1**				0.04^**^	0.04
Sex	−3.44	2.75	−0.08		
Age	0.10	0.04	0.18^**^		
**Step 2**				0.05^**^	0.01
Sex	−3.47	2.75	−0.08		
Age	0.11	0.04	0.19^**^		
Maternal education	−0.42	0.35	−0.08		
**Step 3**				0.07^**^	0.02^**^
Sex	−4.22	2.74	−0.10		
Age	0.10	0.04	0.18^**^		
Maternal education	−0.30	0.35	−0.06		
Cognition	−0.10	0.05	−0.15^**^		
**Step 4**				0.18^***^	0.11^***^
Sex	−3.10	2.62	−0.07		
Age	0.08	0.04	0.13^*^		
Maternal education	−0.44	0.33	−0.08		
Cognition	−0.06	0.04	−0.08		
ADI-R–SI	0.50	0.21	0.20^**^		
ADI-R–communication	0.32	0.25	0.10		
ADI-R–RRB	0.46	0.36	0.09		
**Step 5**				0.18^***^	0.00
Sex	−3.12	2.62	−0.07		
Age	0.07	0.04	0.13		
Maternal education	−0.44	0.33	−0.08		
Cognition	−0.05	0.05	−0.08		
ADI-R–SI	0.48	0.22	0.20^**^		
ADI-R–communication	0.31	0.26	0.10		
ADI-R–RRB	0.46	0.36	0.09		
VABS composite scores	−0.02	0.11	−0.02		

CPRS, conners' parents' rating scales; ADI-R, autism diagnostic interview–revised; SI, social interaction; RRB, restricted repetitive behavior; VABS, adaptive behavior scales.

*** p < 0.001,

** p < 0.01,

* *p < 0.5*.

### Predictors of Adaptive Functioning

An additional five-step hierarchical linear regression analysis using the VABS composite scores as the dependent variable was applied to identify variables that contributed to the variance in the child's adaptive behavior. Independent variables included age and sex in the first step, maternal education in the second step, cognitive scores in the third step, ADI-R SI, Communication and RRB subdomain scores in the fourth step, and CPRS Inattentive, HI and Anxiety scores in the fifth step. As presented in Table 9, the total model explained 44.0% of the variance. In the final step, when all predictors were entered together, age, IQ scores, ADI–R SI and Communication scores, and CPRS inattention scores were significant independent predictors over and above all the other variables entered.

**Table 9:** Linear regression model for the VABS composite scores.

**Variable**	**B**	**SE.B**	**β**	***R*^**2**^**	**Δ*R*^**2**^**
**Step 1**				0.04^**^	0.04^**^
Sex	−1.54	2.09	−0.05		
Age	−0.08	0.03	−0.18^**^		
**Step 2**				0.04^**^	0.00
Sex	−1.52	2.09	−0.05		
Age	−0.08	0.03	−0.18^**^		
Maternal education	0.32	0.26	0.08		
**Step 3**				0.24^***^	0.20^***^
Sex	0.18	1.88	0.01		
Age	−0.07	0.03	−0.17^**^		
Maternal education	0.06	0.24	0.01		
Cognition	0.24	0.03	0.45^***^		
**Step 4**				0.42^***^	0.18^***^
Sex	−0.60	1.67	−0.02		
Age	−0.04	0.02	−0.10^*^		
Maternal education	0.16	0.21	0.04		
Cognition	0.19	0.03	0.37^***^		
ADI-R -SI	−0.54	0.13	−0.29^***^		
ADI-R-communication	−0.43	0.16	−0.18^**^		
ADI-R-RRB	−0.02	0.23	−0.01		
**Step 5**				0.44^***^	0.02^*^
Sex	0.34	1.70	0.01		
Age	−0.05	0.02	−0.11^*^		
Maternal education	0.18	0.21	0.04		
Cognition	0.19	0.03	0.36^***^		
ADI-R–SI	−0.52	0.13	−0.28^***^		
ADI-R–communication	−0.38	0.16	−0.16^**^		
ADI-R–RRB	−0.06	0.24	−0.01		
CPRS Inattention	−0.18	0.06	−0.16^**^		
CPRS HI	0.03	0.06	0.03		
CPRS Anxiety	0.01	0.04	0.01		

VABS, adaptive behavior scales; ADI-R, autism diagnostic interview–revised; SI, social interaction; RRB, restricted repetitive behavior; CPRS, conners' parents' rating scales; HI, hyperactivity/impulsivity.

*** p < 0.001,

** p < 0.01,

* *p < 0.5*.

## Discussion

### Prevalence of Clinically Elevated ADHD and Anxiety Symptoms

The current study found an increased frequency of clinically elevated ADHD symptoms (62.7%) as reported by the parents in a large, well-characterized group diagnosed with ASD. Both clinically elevated 'inattention' symptoms and 'hyperactivity/impulsivity' symptoms were documented at high rates in the ASD group (67 and 57% respectively). These findings were emphasized when comparing the current study's ASD group with the CRS manual's non-clinical group. This was in accordance with our hypothesis. The current study supported previous studies that reported higher ADHD symptom frequencies (16–85%) in ASD (5–7, 9, 25).

In addition, increased frequency of clinically elevated anxiety symptoms (44%) was found in the entire ASD group of the current study. These findings were emphasized when comparing the current study's ASD group with the CRS manual's non-clinical group. The clinically elevated anxiety symptoms are in accordance with previous reports (24, 27) and with our initial hypothesis. Furthermore, the ASD participants with clinically elevated ADHD symptoms in the current study had almost twice the frequency of clinically elevated anxiety symptoms (53%) than the participants with ASD without ADHD symptoms (29.8%). Craig et al. (27) also reported similar findings in a subgroup of ASD and ADHD.

### Parents' and Teachers' Reports

Differences in informant reports of clinically elevated ADHD and anxiety symptoms were also of interest. Parents reported more severe ADHD symptoms, while teachers reported more severe anxiety symptoms. This can be explained as almost one third of the study participants received medication for ADHD, which decreased symptom severity in the structured school setting. In contrast, the school environment provides more stressful social situations, which may lead to more noticeable anxiety symptoms. Therefore, we chose to use only the parental reports for the assessment of comorbidities (see Limitations).

Llanes et al. (36) compared parents' and teachers' reports on ADHD and anxiety symptoms in preschool and school-aged children, and found a low concordance between these informers, with teachers reporting fewer problems overall.

### Clinical Presentation

#### Autism Severity

When looking at ASD with and without specific comorbidities (ADHD and/or anxiety), the subgroup of ASD without comorbid symptoms showed less severe autism symptoms in comparison to the subgroup of ASD with ADHD and/or anxiety. These differences were noted only by parental reports on the ADI-R (socialization, communication, and RRB) with a large effect size, but were not found in the professionals' observations (ADOS). One possible explanation is that children with ASD and comorbidities (ADHD and/or anxiety) exhibit aberrant behaviors at home more than children with ASD without these comorbidities. They may be less attentive, need more support and have more difficulties in change of routines, and consequently are harder to raise. Therefore, they are potentially perceived by their parents as having more severe “autism” than children with only ASD. The professional assessment using the ADOS focuses on specific behaviors in a stimulating and interesting setting and is less affected by ADHD or anxiety-related behavior problems. In addition, the ADOS is a relatively short “snapshot” in real time of a child's observed behaviors in a quiet clinical room 1:1 with a professional. The ADI-R, on the other hand, is based on information given by the child's parents, and includes the child's overall behavior in the “real world,” providing a broader picture than the time-specific ADOS. These findings are in accordance with previous research (14, 37) which suggested that the difference between parental reports and professional assessments in regard to autism severity when clinically elevated ADHD symptoms are present, might be explained by a possible bias of the professional's observation, who may rate ASD symptoms as less severe because some of these symptoms are attributed to the co-morbid ADHD symptoms. These findings were in partial accordance with our hypothesis, as having comorbidities was associated with more severe autism symptoms only as reported by the parents, and having more than one comorbidity was not associated with greater autism severity.

#### Adaptive Skills

Regarding adaptive skills, having ASD with clinically elevated ADHD symptoms was associated with greater impairments in socialization adaptive skills. Only the subgroup with ASD and the two comorbidities was associated with poorer daily living adaptive skills. These findings partially supported our hypothesis, as not all the adaptive skills domains were associated with the comorbidities. However, having two comorbidities was associated with more adaptive functioning domains, albeit with a medium effect size. The current research is in line with previous findings that individuals with ASD and ADHD have a tendency toward greater impairments in adaptive functioning in all the VABS subdomains as compared to those with only ASD (2, 37, 38).

#### Cognitive Ability

Although ASD with clinically elevated symptoms of ADHD and/or anxiety was associated with lower adaptive skills functioning, surprisingly and not in accordance with our hypothesis, it was not associated with lower cognitive ability. Other studies found more impairments in verbal and non-verbal abilities, spatial working memory, response inhibition and executive functions in ASD with ADHD (37, 38). A lack of association between clinically elevated ADHD and/or anxiety symptoms and cognitive ability may be explained by the notion that cognition is an innate ability, which theoretically is not affected by comorbidities. The diagnosis center's team has a great deal of experience working with children with ASD, and it may be that they were able to help the children reach their best potential in terms of cognitive ability.

Regarding our third aim, in accordance with our hypothesis, the severity of clinically elevated ADHD and anxiety symptoms were associated with more severe autism symptoms and poorer overall functioning.

### Predictors of ADHD and Anxiety Symptom Severity

Our Forth aim was to assess which variables added to the severity of Inattention, Hyperactivity/Impulsivity, and anxiety symptoms individually. For Inattention symptom severity, the regression model explained 12.3% of the variance. Being a female and having lower adaptive skills scores were greater predictors of higher severity of Inattention symptoms, beyond the contribution of age, parental educational attainment, cognition, and autism severity. It is possible that lower functioning is associated with the severity of inattention as perceived by parents. The finding regarding females is important and should be further examined in future studies on inattention in a subgroup of females with ASD. For Hyperactivity/impulsivity symptom severity, the regression model explained 18.5% of the variance. Being older, having greater cognitive abilities and more severe RRB symptoms were greater predictors of higher severity of HI symptoms, beyond sex, parental educational attainment, adaptive skills, and autism severity in the social interaction and communication domains. It may be that ADHD is easier to diagnose in older children with greater cognitive abilities. In addition, RRB symptoms may overlap with HI symptoms (i.e., motor stereotypies, repetitive behaviors), and therefore children with more RRB symptoms will be perceived as having more HI symptoms. For anxiety symptom severity, the regression model explained 21% of the variance. Being older and having more severe social impairments were greater predictors of higher anxiety scores, beyond sex, parental educational attainment, adaptive skills, and autism severity in the communication and RRB domains. This may reflect that anxiety symptoms are more easily recognized in older children. Social impairments and anxiety symptoms often overlap or augment each other. Overall our hypothesis regarding the association between the comorbidities and cognitive level, autism severity and adaptive functioning was partially supported, as each comorbidity was associated with different factors.

### Predictors of Adaptive Functioning

Adaptive functioning refers to behaviors critical in everyday life in terms of communication, socialization, daily living, and motor skills. Adaptive skills are one of the most important measures in evaluating functioning in ASD, as they reflect the generalization of acquired skills in everyday life. Therefore, we thought it important to identify the variables that may predict overall adaptive skills by using regression models as outlined in our Fifth aim. For overall level of adaptive skills, the regression model explained 44% of the variance. Younger age, better cognition, less severe social and communication impairments, and less severe inattention symptoms, all as reported by parents were greater predictors of better overall adaptive skills, beyond sex, maternal education, autism severity in the RRB domain, and the severity of HI and anxiety symptoms. These findings suggest that in ASD, in addition to the role of cognitive ability and autism severity, the severity of inattention symptoms adversely relates to the overall adaptive functioning. Interestingly, only the severity of Inattention and not that of Hyperactivity/impulsivity or anxiety contributed to lower adaptive functioning, over and above other ASD-related characteristics. These findings partially support our hypothesis, as only inattention symptoms but not HI and anxiety symptoms predicted the level of adaptive skills.

### Strengths and Limitations

The current study has several strengths. One is the large sample size of a well-characterized ASD sample. The sample contained both males and females. There were no significant differences between the four examined groups in any demographic parameters. The assessment of ASD was conducted using gold standard tests (ADI-R; ADOS) and ADHD and anxiety symptomatology were obtained using standardized questionnaires (CRS). The use of these comprehensive assessments promotes a broader understanding of the clinical presentation in the subgroup diagnosed with ASD and ADHD and/or anxiety.

This study has several limitations. There is a lack of a control group of children with neither ASD nor comorbidities; in the future, it will be important to design such a study. In addition, there were missing data points for some participants in the cognitive test scores (28) and the ADOS severity scores (5). Severity of symptoms of ADHD and anxiety was based on standardized questionnaires, which allowed for the quantification of a continuous variable, and not on the clinical diagnosis of these disorders. We compared parents' and teachers' CRS scores, but used only the parental CRS to examine the relationship between the severity of ADHD and anxiety symptoms and the levels of autism severity, cognitive ability and adaptive skills. Since more than a quarter of our study participants were previously diagnosed with ADHD, and many were treated with medications during the school day, the parents' reports were used to avoid the potential effects of medication on the reported severity of the ADHD symptoms. Previous research has used only parental CRS as well (25). Indeed, teachers' reports were less severe than the parents' in terms of ADHD symptoms. However, the teachers reported more severe anxiety symptoms as compared to the parents, and this was not reflected in the analyses that were based on parental reports. Finally, the anxiety subscale of the CSR was used to assess anxiety, instead of a specific anxiety questionnaire.

### Study's Significance

The novelty of the current study is that it is the first to examine the relationship between having ADHD and/or anxiety in ASD and the clinical presentation in autism severity, adaptive skills and cognitive ability. In addition, the current study investigated for the first time which variables are associated with symptoms of inattention, hyperactivity/impulsivity and anxiety in ASD. Finally, the study addressed which variables may comprehensively affect adaptive skills in ASD by considering the contribution of ADHD and anxiety co-morbidities in addition to more traditional variables, such as the child's age, sex, cognitive ability, and autism severity. This led to the unique finding that only a specific ADHD symptom—inattention—contributes to lower adaptive functioning in ASD.

This study has theoretical significance. The very high frequency of ADHD symptoms in over two thirds of the ASD group suggests that some of the symptoms of both disorders may overlap. ASD and ADHD are described with similar brain abnormalities in specific regions, including the medial frontal and prefrontal cortex, which play an important role in executive functions (39), and reduced activation in the striato-thalamic region, prefrontal, and parietal cortex (40). Our finding of the significant co-occurrence of ADHD symptoms in ASD, along with the CNS abnormalities in both disorders, implies a common neurobiological origin. Significant anxiety symptoms may aggravate certain ASD symptoms, such as social withdrawal, avoidance of exposure to different stimuli, and increased ritual/stereotypic behaviors, which may affect overall functioning. Therefore, it is possible that there may be overlap between symptoms of ASD and anxiety. Structural and functional abnormalities of the amygdala were found in ASD (41), suggesting more extensive impairment and more biological insults, which may manifest in a more severe clinical presentation.

### Implications and Future Research

The study has several clinical implications. In light of the finding that ADHD and anxiety symptoms are related to poorer adaptive behaviors and to more severe autism symptoms as perceived by the parents, it is highly important during the ASD diagnostic process to assess ADHD and anxiety symptomatology. Since having ADHD and/or anxiety symptoms with ASD is associated with parents' negative perceptions of their child's functioning, parents should be provided with support and guidance.

In addition, since many children in this study were diagnosed first with ADHD and only later with ASD, it may be that the professionals were influenced by the dominant ADHD symptoms and they associated the social communication and RRB symptoms with the ADHD diagnosis. A very important takeaway for developmental behavioral pediatricians, pediatric neurologists and child psychiatrists is to consider a possible diagnosis of ASD when a young child exhibits significant ADHD symptomatology and when there are social difficulties and evidence for sensory or repetitive restrictive behaviors too.

In light of the study's findings, it will be important to diagnose these comorbidities early on in children with ASD, and to explore effective medical and behavioral interventions to reduce the impact of these comorbidities, thereby hopefully leading to improved functioning.

Future studies should further explore the clinical presentation in females. In addition, it would be interesting to explore the impact of medication and specific intervention programs to lower anxiety on the clinical presentation of children with ASD and ADHD and/or anxiety symptoms. It is also important to investigate genetic and imaging differences between children with ASD alone, ASD and ADHD, and ASD, ADHD and anxiety.

## Author Contributions

EA, EB-I, and DZ contributed to the design and implementation of the research, to the analysis of the results, and to the writing of the manuscript.

### Conflict of Interest Statement

The authors declare that the research was conducted in the absence of any commercial or financial relationships that could be construed as a potential conflict of interest.
